# A Safety and Tolerability Study of Thin Film Freeze-Dried Tacrolimus for Local Pulmonary Drug Delivery in Human Subjects

**DOI:** 10.3390/pharmaceutics13050717

**Published:** 2021-05-13

**Authors:** Sawittree Sahakijpijarn, Moeezullah Beg, Stephanie M. Levine, Jay I. Peters, Robert O. Williams

**Affiliations:** 1 Division of Molecular Pharmaceutics and Drug Delivery, College of Pharmacy, The University of Texas at Austin, Austin, TX 78712, USA; sawittree.willy@utexas.edu; 2 Department of Pulmonary and Critical Care, The University of Texas Health Science Center at San Antonio, San Antonio, TX 78229, USA; beg@uthscsa.edu (M.B.); LevineS@uthscsa.edu (S.M.L.)

**Keywords:** lung transplantation, immunosuppressant, tacrolimus, thin film freezing, dry powder inhalation, nebulization, safety, tolerability

## Abstract

Due to the low and erratic bioavailability of oral tacrolimus (TAC), the long-term survival rate following lung transplantation remained low compared to other solid organs. TAC was reformulated and developed as inhaled formulations by thin film freezing (TFF). Previous studies reported that inhaled TAC combined with 50% *w/w* lactose (LAC) was safe and effective for the treatment of lung transplant rejection in rodent models. In this study, we aimed to investigate the safety and tolerability of TFF TAC-LAC in human subjects. The formulation can be delivered to the lung as colloidal dispersions after reconstitution and as a dry powder. Healthy subjects inhaled TAC-LAC colloidal dispersions at 3 mg TAC/dose via a vibrating mesh nebulizer in the first stage of this study and TAC-LAC dry powder at 3 mg TAC/dose via a single dose dry powder inhaler in the second stage. Our results demonstrated that oral inhalation of TAC-LAC colloidal dispersions and dry powder exhibited low systemic absorption. Additionally, they were well-tolerated with no changes in CBC, liver, kidney, and lung functions. Only mild adverse side effects (e.g., cough, throat irritation, distaste) were observed. In summary, pulmonary delivery of TFF TAC-LAC would be a safe and promising therapy for lung transplant recipients.

## 1. Introduction

Lung transplantation has become a life-saving treatment option for patients with end-stage lung diseases. The number of lung transplants has steadily increased over the years worldwide [1]. According to the 2020 Registry of the International Society for Heart and Lung Transplantation, more than 4500 lung transplants were performed annually [2]. Almost 70,000 lung transplant cases have been reported worldwide between 1992 and 2018 [2]. Although a median survival has been improved from 4.7 to 6.7 years over the last decade [3], long-term survival remained the lowest compared to other solid organ transplants [4]. The long-term survival rate is limited by acute and chronic rejection, infectious complications, drug toxicities, and malignancies [4].

Maintenance immunosuppression is currently used to prevent acute and chronic rejection. The current drug regimen for lung transplantation consists of a combination of three orally administrated drugs, including a calcineurin inhibitor (e.g., cyclosporin or tacrolimus), anti-metabolite (e.g., mycophenolate or azathioprine), and corticosteroids [4].

Tacrolimus (TAC) is a potent immunosuppressive agent and acts by inhibiting T-lymphocyte activation and proliferation [5]. TAC is the most commonly used agent for maintenance immunosuppression since several studies have shown a better outcome, including a lower incidence of acute rejection and an improvement of long-term survival rates, compared to cyclosporin [6,7,8,9,10]. Currently, TAC has been available as an intravenous infusion and in oral dosage form [11]. Unfortunately, the oral formulation poses many issues, namely limited and widely variable bioavailability [12]; multiple drug interactions; and significant systemic adverse effects such as nephrotoxicity, neurotoxicity, worsening hypertension, and new-onset diabetes mellitus [13], which make this route of administration less than ideal. To address these issues, pulmonary drug delivery offers a more viable and safer route of administration. It could provide a higher concentration of the drug within the lungs, while limiting systemic absorption, and result in a potentially lower incidence of adverse effects.

Novel formulations of TAC have been developed by thin film freezing (TFF) technology. TFF is a bottom-up particle engineering technology that can modify physicochemical properties of a drug such as particle size, surface characteristics, morphology, and crystallinity [14]. TAC was combined with lactose (LAC) and then dissolved in the organic solvent mixture. The drug and excipient solutions were dropped and frozen on a cryogenic drum. The frozen discs were dried by lyophilization. The ultra-rapid freezing rate can prevent the crystallization of drugs and minimize the particle aggregation of dissolved solute, thereby forming nanostructured aggregates brittle matrix powder [14].

Previous studies demonstrated that the novel formulation can be delivered to the lung using both a nebulizer and a dry powder inhaler (DPI) [15,16]. For nebulization, TFF TAC-LAC powder can be dispersed as colloidal dispersions in aqueous media and subsequently atomized using a vibrating mesh nebulizer [15]. The in vitro aerodynamic testing using a next generation pharmaceutical impactor demonstrated that the droplet of nanostructured dispersion generated by an Aeroneb^®^ Pro vibrating mesh nebulizer exhibited an optimal aerosol performance (4.06 µm mass median aerodynamic diameter (MMAD) and a 46.1% fine particle fraction (FPF), 50.5% emitted dose) [17]. Particle size analysis by laser diffractometer was used to investigate the physical stability of TFF TAC-LAC colloidal dispersion over time. The colloidal dispersion remained as a monodisperse distribution with a mean particle diameter of 300 nM at 15 min after reconstitution [17]. Hence, it was suggested to complete drug administration via nebulization within 15 min after reconstitution.

In addition to nebulization, TFF TAC-LAC dry powder has a high surface area, high porosity, and low density, which provide benefits to powder dispersibility and aerosolization. In situ brittle matrix powder can be sheared and dispersed by a shear force from a passive DPI [16,18]. The in vitro aerodynamic testing showed that TFF TAC-LAC dry powder exhibited optimal aerosol performances via a Handihaler^®^ (2.7 ± 0.3 µm MMAD, 68.7 ± 5.9% FPF of delivered dose) [18]. Additionally, XRD diffractograms showed TAC and LAC were amorphous after the process. TFF TAC-LAC showed a faster dissolution rate than unprocessed TAC powder due to a higher solubility of amorphous TAC and a higher surface area of TFF powder [15,18]. Moreover, the formulation was chemically stable with no change in drug potency. The amorphous TAC was also physically stable and retained its ability to achieve supersaturation in simulated lung fluid throughout the 3-month period [19].

The in vivo pharmacokinetic studies in rodent models demonstrated that the novel formulation can achieve a mean peak transplanted lung concentration of 399.8 ± 29.2 ng/g after a single-dose administration of TFF TAC-LAC dispersion [17], which is higher than previously reported therapeutic concentrations necessary to provide significant immunosuppression in the lungs [20]. Additionally, the mean peak blood concentration after a single-dose administration was 4.88 ± 1.6 ng/mL [17]. It was found that pulmonary administration of TAC exhibited a higher lung-to-blood ratio compared to oral administration (59:1 vs. 9:1, respectively). This indicates the benefit of pulmonary administration of TAC, which can minimize system drug levels and system side effects.

Moreover, our research groups have studied the efficacy and safety of inhaled TFF TAC-LAC in rats. Das et al. demonstrated that inhaled TFF TAC-LAC showed a similar efficacy as systemic TAC in preventing rejection in the orthotopic rat lung transplant model [21]. Additionally, lower systemic TAC trough levels were detected in inhaled TFF TAC-LAC treated rats compared to intramuscular-treated rats [21]. Another study also reported that TFF TAC-LAC can be delivered to the rat lungs and exhibited high concentrations for 28 days without histologic changes in the lung and significant changes in CBC, liver function, or renal function [19]. Systemic TAC concentration after 24 h administration of TAC dispersion was lower than toxicity level [19].

According to several studies, pulmonary delivery of TFF TAC-LAC is safe and has low potential to induce toxic side effects in rodent models. However, to our knowledge, there has been no study evaluating the safety and tolerability of TFF TAC-LAC in humans. Therefore, we sought to evaluate the use of TFF TAC-LAC in healthy human volunteers. The objective of our study was to study the safety and tolerability of this novel formulation of TAC in healthy volunteers.

## 2. Materials and Methods

### 2.1. Materials and Sample Preparation

**Figure 1:**
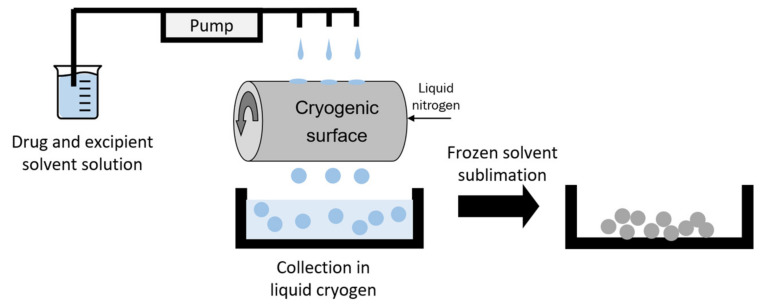
Illustration of TFF process. Modified with permission from MDPI [22].

TAC was purchased from Teva Pharmaceutical Industries (Petah Tikva, Israel), while lactose monohydrate was purchased from Fisher Scientific (Pittsburgh, PA, USA). TFF TAC-LAC was prepared by thin film freezing, as shown in Figure 1. TAC and LAC (50/50 *w/w*) was dissolved at 0.75% solid content in acetonitrile/water (60/40 *v/v*). The solution was dropped on a cryogenic drum that is filled with liquid nitrogen. By an ultra-rapid freezing rate, the droplets were frozen immediately and became frozen discs. The frozen samples were collected in a container filled with liquid nitrogen. Then, the frozen samples were transferred and dried in a lyophilizer. The primary drying was set at −40 °C for 20 h. Then, the shelf temperature was gradually increased to 25 °C over 20 h. Finally, the samples were secondary dried at 25 °C over 20 h. The vacuum pressure was controlled at 100 mTorr during the whole drying cycle.

### 2.2. Study Design and Population

A pilot study designed to test safety, side effect profile, and pharmacokinetics of a novel formulation of inhaled TFF TAC-LAC in healthy adults was conducted at Joe R. and Teresa Lozano Long School of Medicine, University of Texas Health San Antonio, Texas. Twenty healthy volunteers (10 males and 10 females) between the ages of 18–55 years were included in the study after obtaining approval from Institutional Review Board (Protocol number: HSC20100004H). Informed consent was obtained from all volunteers. The privacy rights of human subjects are always observed. The study was performed in two stages to study two different methods of delivering inhaled TFF TAC-LAC. Inhalation of TFF TAC-LAC via a nebulizer and a DPI were studied in the first and second stages, respectively.

#### 2.2.1. Stage I—Pulmonary Administration of TFF TAC-LAC Colloidal Dispersion via a Vibrating Mesh Nebulizer

Twenty adult subjects (10 males and 10 females) were enrolled for the first stage this study. TFF TAC-LAC powder [3 mg of TAC + 3mg of LAC] was dispersed in sterile water for injection. A colloidal dispersion of TFF TAC-LAC (3 mg TAC/dose) was atomized using an Aeroneb Pro^®^ nebulizer (Aerogen, Galway, Ireland). The Aeroneb Pro^®^ is a lower energy piezoelectric nebulizer that is also called a micropump. Inside the nebulizer is a dome that vibrates the dispersion 1000 times per second. Each droplet contains hundreds of amorphous 0.15-micron particles. Due to their extremely small size, the droplets allow the TAC particles to be delivered to the distal airways.

#### 2.2.2. Stage II—Pulmonary Administration of TFF TAC-LAC Inhalable Powder via a DPI

In the second stage of the study, 10 healthy adult subjects (5 males and 5 females) were randomly selected from the previously enrolled 20 healthy volunteers for the first stage of the study. Subjects were between the ages of 18–55 years. Six milligrams of TFF TAC-LAC as a brittle matrix powder (3 mg of TAC + 3 mg of LAC) were filled in an HPMC capsule. Subjects inhaled 6 mg of TFF TAC-LAC powder (3 mg TAC/dose) using a single dose passive DPI (Handihaler^®^, Boehringer Ingelheim GmbH, Ingelheim am Rhein, Germany).

### 2.3. Exposures, Covariates and Outcomes

#### 2.3.1. Outcomes

The subjects were asked to maintain a symptom diary to record symptoms from 1 h to 24 h after inhalation. The symptoms recorded were a cough, an abnormal taste, shortness of breath, any abnormal throat sensations, and chest discomfort. These were rated on a scale of 0–5 with 0 being the absence of symptoms and 5 being the most severe. For the TFF TAC-LAC DPI, subjects were also asked to record symptoms they experienced during inhalation.

Spirometry and plasma TAC levels were obtained at 1 h post-inhalation of either nebulized or inhaled dry powder TAC. Twenty-four hours after inhalation, 10 of the 20 subjects in the nebulized TFF TAC-LAC group and all 10 subjects in the TFF TAC-LAC DPI group underwent repeat spirometry.

Plasma trough TAC levels were drawn 24 h after inhalation to assess systemic absorption. TAC levels were measured using a commercially available assay for human whole blood (PRO-Trac II FK 506 ELISA kit, DiaSorin Inc., Saluggia, Italy).

#### 2.3.2. Ascertainment of Other Covariates

At baseline, the subjects performed spirometry, and had a baseline TAC level (determined by immunoassay), a comprehensive metabolic profile (CMP), and a complete blood count (CBC) drawn. CMP and CBC were also drawn 1 h post-inhalation.

### 2.4. Statistical Analysis

Categorical variables were presented as frequencies with percentages. Continuous variables were presented as mean with standard deviation. The statistical significance of experimental results was conducted using the two-sample t-test in JMP 10 (SAS Institute, Cary, NC, USA). The alpha level was set at 0.05.

## 3. Results

### 3.1. Pulmonary Administration of TFF LAC-LAC Colloidal Dispersion via a Nebulizer

The 20 patients who received nebulized TFF TAC-LAC were 35.9 ± 15.3 years old and had a mean duration of inhalation of 9.3± 3.8 min. The baseline and 24 h post-inhalation laboratory values are shown in Table 1. All subjects had normal baseline laboratory values. Laboratory values obtained 24 h after inhalation of a 3 mg TAC/dose did not show any significant changes. The baseline, 1 h post-inhalation, and 24 h post-inhalation spirometry values are shown in Table 2. The spirometry values for all subjects at baseline were within expected normal limits for their age. The spirometry values 1 h and 24 h after inhalation showed very minimal changes that were not statistically significant. Mean plasma TAC levels were 4.64 ng/mL ± 2.77 1 h after inhalation and were undetectable 24 h after inhalation. 

Patients were asked to keep a symptom diary of the frequency of symptoms along with the severity on a scale of 0–5. Table 3 shows the adverse effects after administration of nebulized TAC-LAC. The most commonly reported symptom 1 h post-inhalation of nebulized TAC-LAC was an abnormal taste, which was noted by eight subjects, with a mean severity score of 0.55 (from a scale of 0–5). Other frequently reported symptoms included an abnormal throat sensation in three subjects, a cough in one subject, and shortness of breath in one subject. The most common symptom at 24 h post-inhalation was a mild abnormal taste, reported by eight subjects, that resolved by 48 h.

### 3.2. Pulmonary Admistration of TFF LAC-LAC Inhalable Powder via A DPI

The mean age of the 10 subjects who were enrolled to receive TFF TAC-LAC inhalable dry powder was 35.13 ± 9.8 years. All subjects had normal lung exams and normal spirometry at baseline and completed the inhalation via a passive DPI with a single inspiratory effort. Table 4 shows the baseline and 24 h post-inhalation laboratory values, while Table 5 shows the baseline, 1 h post-inhalation, and 24 h post-inhalation spirometry values after inhalation of a 3 mg TAC/dose. There was no significant difference in laboratory values between the baseline and 24 h after inhalation. Likewise, spirometry values obtained 1 h post-inhalation and 24 h post-inhalation were unchanged from baseline for all 10 subjects. Mean blood TAC levels were undetectable at 1 h in 6/10 subjects and were low in 4/10 subjects (2.5 ± 0.5 ng/mL).

Table 6 shows the adverse effects after administration of TAC-LAC dry powder for inhalation. During inhalation, the most commonly reported symptom was a cough and distaste, reported by eight subjects, followed by throat irritation (three subjects). One hour post-inhalation, all 10 subjects experienced distaste in their mouth, and nine subjects reported a cough. No symptoms were reported 24 h post-inhalation. Subsequent testing with the same subjects demonstrated that rinsing and gargling post-inhalation resulted in minimal throat irritation and cough.

## 4. Discussion

Despite undeniable evidence that chronic lung allograft dysfunction is significantly associated with increased morbidity and mortality [1,23] and growing recognition of the role of immunosuppression in the prevention of chronic lung allograft dysfunction [24], current treatment options remain limited. TAC is the most commonly used immunosuppressive agent. Since it is a drug with a narrow therapeutic index [25], the long term success of TAC is associated with significant systemic adverse events including kidney dysfunction and neurotoxicity [26,27].

Lower blood concentration may affect the efficacy of treatment and subsequently develop a rejection episode, while higher concentration may cause toxicities such as nephrotoxicity and/or neurotoxicity [25]. Approximately 23.3% of lung transplant recipients suffer from severe renal dysfunction at 1 year, and 55.4% will have evidence of renal dysfunction 5 years post-transplant [28]. This is most commonly related to chronic administration of calcineurin inhibitors such as cyclosporine and TAC [26,29]. Furthermore, individuals who develop renal dysfunction after lung transplantation have consistently demonstrated higher mortality [29,30]. Therefore, it is imperative to develop a method involving direct delivery of the drug to the lung and limit systemic toxicity.

Our study showed that pulmonary delivery of TFF TAC-LAC is safe and well-tolerated by healthy adults free of any disease. Several previous studies reported that there were some relationships between TAC concentration and toxicity in transplant patients [31,32,33]. The mean plasma TAC level 1 h post-inhalation in the nebulized TAC-LAC group was significantly below the toxic range and below the systemic level measured at the trough in oral dosing [19,27]. Importantly, the plasma TAC levels were undetectable at 24 h in all subjects.

Our results agree with the literature. Schrepfer et al. compared oral and inhaled TAC in the orthotopic rat tracheal transplantation model and noted that low blood levels were detected after delivery of inhaled TAC [34]. Systemic side effects were only observed with oral TAC. Additionally, they reported that both routes were effective in preventing acute rejection and chronic obliterative airway disease. Recently, Das et al. also reported that TAC trough levels in the kidneys and plasma following pulmonary administration of TFF TAC-LAC were lower than an injectable administration of TAC in an orthotopic rat lung transplant model [21]. Another study from Watts et al. also investigated the safety and systemic elimination of TAC following pulmonary administration of nebulized TFF TAC-LAC once daily for 28 consecutive days in Sprague Dawley rats. It was reported that systemic TAC concentration at 24 h after the final dose was 1.0 ± 0.5 ng/mL, which is well below the clinically accepted trough concentrations (5–15 ng/mL) for maintenance therapy [19]. Therefore, these results could have a significant clinical utility as an immunosuppressive agent to lower the risk of renal dysfunction, especially in patients with baseline chronic kidney disease. Furthermore, it could also be used to replace oral TAC in patients who demonstrate a post-transplant rise in serum creatinine, to prevent or slow the progression of renal dysfunction.

There were no changes in CBC and renal or hepatic function panels at 1 h and 24 h after inhalation when compared to the baseline laboratory data. Additionally, inhaled TAC is well-tolerated in human subjects with minimal side effects immediately post-inhalation and without a significant change in the spirometry parameters. The most commonly noted side effects post-inhalation were mild and included an abnormal taste, a cough, and throat irritation. The safety profile of TFF TAC-LAC in human subjects from our study corresponds to previous animal studies [15,19]. It has demonstrated that TFF TAC-LAC can be effectively delivered to the lung without causing any damage to the lung tissue [15,19]. Pulmonary administration of once-daily nebulized TFF TAC-LAC to mice for 28 days showed no evidence of histological changes or inflammation in the lungs and there were also no significant alterations in hematocrit, white count, platelet count, liver function, or renal function [19].

Interestingly, the incidence of post-inhalation cough following dry powder inhalation was higher than nebulization. Post-inhalation cough is a side effect that has been reported in several products including aqueous base aerosols, pressurized metered-dose inhaler, and DPI. Our observation agrees with the case of tobramycin inhalation solution (TIS) and tobramycin inhalation powder (TIP) [35,36]. Cough incidence was higher in patients receiving TIP in comparison to the TIS group [35,36]. The differences in a delivery mechanism and inspiratory flow profiles when inhaling through a nebulizer and a DPI are possibly associated with cough incidence [37]. Aerosols of drug solutions/dispersion were delivered using a nebulizer over 10–20 min, while a DPI delivers powder as a bolus in 1–2 inhalations [35]. Therefore, there are more drug particles depositing on the pulmonary epithelium for a given breath when inhaling dry powder [37]. Besides, more forceful and rapid inhalation is generally required to disperse and deliver powder effectively through a DPI [37]. The rapid inhalation can lead to larger inhaled volumes and an increase in lung recoil, thereby increasing the potential of cough [38]. It was suggested that the use of a higher resistance inhaler, which is required for a lower inspiratory flow rate, may minimize the cough incidence [39]. This is possible for inhaled TAC powder prepared using TFF since TFF TAC-LAC showed consistent aerosol performance over the different flow rates, indicating low flow rate dependency [18]. Although a few side effects were observed in our study, most of them were mild scores and were improved over time after administration. Therefore, it is unlikely that these will be a significant deterrent for use in lung transplant patients.

The findings in our study add to the growing body of literature supporting the use of inhaled TAC as a safe delivery method in lung transplant patients. Although our current study did not include the efficacy test in humans, inhaled TAC has been shown to be an effective immunosuppressive agent in several studies. Deuse et al. exposed different levels of aerosolized TAC to human airway epithelium in cell cultures [40]. They demonstrated that inhaled TAC was able to penetrate the lungs and then moves through the epithelium to the sub-basilar space, where it inhibited lymphocyte activation. The level of inhibition was dose-dependent with higher levels leading to higher immunosuppression [40]. Ide et al. performed a comparison between systemic TAC (delivered intra-muscularly) and inhaled TAC (micronized powder formulation) in rats that had undergone a single orthotopic lung transplant [20]. The study demonstrated that high dose inhaled TAC was just as effective in preventing acute and chronic graft dysfunction as oral TAC but with a significantly reduced number of proliferating antigens produced by bronchial lymphoid tissue as compared to the systemic TAC group [20]. Similarly, TFF TAC-LAC formulation has also been shown to effectively interact with and inhibit lymphocytes using in vitro cellular assays such as mixed leukocyte culture and phytohemagglutinin assays [41]. According to the multiple animal lung transplant models, inhaled TAC is an effective strategy for preventing both acute and chronic rejection and may have a possible role in preventing or treating rejection in the future. Larger prospective studies in humans to assess the clinical efficacy of the inhaled formulation as an immunosuppressive agent need to be pursued.

## 5. Conclusions

Our study demonstrated that both the nebulization of TFF TAC-LAC colloidal dispersion and dry powder inhalation of TFF TAC-LAC powder were well-tolerated and safe methods for targeted drug delivery to the lung. Inhaled TAC had similar pharmacological effects in humans as it did in animal models. The inhalation route offers the opportunity of delivering an immunosuppressant directly to the target site, thereby lowering systemic absorption. Therefore, inhaled TAC should be considered a viable therapeutic option for patients with lung conditions such as lung transplantation or patients with other lung diseases requiring immunosuppression.

## Figures and Tables

**Table 1:** Baseline and 24 h post-inhalation laboratory data with nebulized TAC-LAC (3 mg TAC/dose).

Lab Parameter	Pre-Inhalation (N = 20)	24 h Post-Inhalation (N = 20)
Hemoglobin (Hgb) g/dL	13.5 ± 1.5	13.4 ± 1.5
White Blood Cell (WBC) × 10^9^/L	6.0 ± 1.9	6.2 ± 1.4
Potassium mEq/L	3.8 ± 0.3	3.9 ± 0.2
Blood Urea Nitrogen (BUN) mg/dL	11.7 ± 3.1	11.0 ± 3.1
Creatinine mg/dL	0.76 ± 0.18	0.78 ± 0.22
Aspartate Aminotransferase (AST) IU	25.2 ± 19.5	31.1 ± 8.3

**Table 2:** Baseline, 1 h, and 24 h post-inhalation spirometry values with nebulized TAC-LAC colloidal dispersion (3 mg TAC/dose).

Variable	Pre-Inhalation (N = 20)	1 h Post-Inhalation (N = 20)	24 h Post-Inhalation (N = 10)
FEV1 (L)	3.34 ± 0.69	3.31 ± 0.71	3.31 ± 0.80
FVC (L)	4.16 ± 0.83	4.08 ± 0.82	4.09 ± 0.92
Ratio	0.80 ± 0.06	0.81 ± 0.06	0.81 ± 0.06
FEF 25–75% (L/sec)	3.23 ± 1.13	3.33 ± 1.08	3.29 ± 1.21

**Table 3:** Adverse effects following pulmonary dosing with nebulized TAC-LAC colloidal dispersion (3 mg TAC/dose).

Symptoms	% Number of Patients (N = 20)
1 h following inhalation	
• Cough	5 (1)
• Shortness of breath	5 (1)
• Abnormal throat sensation	15 (3)
24 h following inhalation	
• Abnormal taste	20 (16)
48 h following inhalation	
• Abnormal taste and throat sensation	0 (0)

**Table 4:** Baseline and 24 h post-inhalation laboratory data with TAC-LAC dry powder for inhalation (3 mg TAC/dose).

Lab Parameter	Pre-Inhalation (N = 10)	24-h Post-Inhalation (N = 10)
Hemoglobin (Hgb) g/dL	13.4 ± 1.6	13.5 ± 1.5
White Blood Cell (WBC) × 10^9^/L	6.2 ± 1.9	6.5 ± 1.4
Potassium mEq/L	4.0 ± 0.3	3.9 ± 0.2
Blood Urea Nitrogen (BUN) mg/dL	10.7 ± 3.2	11.0 ± 3.1
Creatinine mg/dL	0.71 ± 0.17	0.74 ± 0.21
Aspartate Aminotransferase (AST) IU	25.6 ± 10.5	29.1 ± 8.3
Alanine Aminotransferase (ALT) IU	24.5 ± 10.2	25.4 ± 10.6
Alkaline Phosphatase IU	65.4 ± 18.4	64.5 ± 16.3

**Table 5:** Baseline, 1 h, and 24 h post-inhalation spirometry values with TAC-LAC dry powder for inhalation (3 mg TAC/dose).

Variable	Pre-Inhalation (N = 10)	1 h Post-Inhalation (N = 10)	24 h Post-Inhalation (N = 10)
FEV1 (L)	3.36 ± 0.70	3.35 ± 0.79	3.37 ± 0.81
FVC (L)	4.16 ± 0.83	4.18 ± 0.80	4.08 ± 0.90
Ratio	0.8 1± 0.06	0.80 ± 0.05	0.82 ± 0.06
FEF 25–75% (L/sec)	3.30 ± 1.16	3.31 ± 1.09	3.30 ± 1.19

**Table 6:** Adverse effects following pulmonary dosing with TAC-LAC dry powder for inhalation (3 mg TAC/dose).

Symptoms	% Number of Patients (N = 10)
During inhalation	
• Cough	80 (8)
• Throat irritation	30 (3)
• Distate	80 (8)
1 h following inhalation	
• Cough (mild)	90 (9)
• Distaste (mild)	100 (10)
24 and 48 h following inhalation	0 (0)

## Data Availability

The data presented in this study are available on request from the corresponding author. The data are not publicly available, but are maintained and available upon request.
